# Characterization of the major latex protein gene family and functional analysis of *GmMLP9* under salt stress in soybean

**DOI:** 10.3389/fpls.2026.1887220

**Published:** 2026-08-03

**Authors:** Benshuai Yu, Rongfan Zheng, Ran Xu, Tongfei Tan, Wei Liu, Xiao Wang, Yanwei Zhang, Lifeng Zhang, Wei Li, Caijie Wang, Xiaoyan Zhang, Haiying Dai, Yubin Wang

**Affiliations:** 1 Crop Research Institute, Shandong Academy of Agricultural Sciences, Jinan, Shandong, China; 2 College of Agronomy, Shandong Agricultural University, Taian, Shandong, China; 3 College of Life Sciences, Qingdao Agricultural University, Qingdao, Shandong, China

**Keywords:** genome analysis, major latex protein, overexpression, salt stress, soybean

## Abstract

**Introduction:**

The major latex proteins (MLPs) belong to the Bet v 1/PR-10 protein family, which is widely distributed among in higher plants and crucial for plant growth, hormone signaling, and abiotic stress responses. While *MLP* genes have been characterized in various plant species, the soybean *MLP* gene family and its contributions to salt tolerance remain poorly understood.

**Methods:**

In this study, we integrated soybean genomic and transcriptomic data to comprehensively identify *MLP* family members using bioinformatics approaches. We systematically analyzed their physicochemical properties, phylogenetic relationships, gene structures, collinearity, promoter cis-regulatory elements, and expression profiles under NaCl and ABA treatments. Additionally, we validated the function of the candidate gene *GmMLP9*.

**Results:**

A total of 41 *MLP* family genes were identified in the soybean genome, with protein lengths ranging from 125 to 319 amino acids. Phylogenetic analysis revealed that soybean *MLP* proteins clustered into three distinct subgroups: I, II, and IV. Collinearity analysis revealed 16 pairs of collinear genes within this family, which were predominantly distributed across various chromosomes, with higher densities observed on chromosomes 7 to 9, 15, and 17, all arising from segmental duplications. Selection pressure analysis indicated that the duplicated genes primarily experienced purifying selection throughout evolution. Promoter analysis identified numerous cis-regulatory elements associated with stress and hormone responses. Expression pattern analysis demonstrated that most *MLP* genes exhibited elevated transcript levels in roots. Under NaCl and ABA treatments, 19 candidate *MLP* genes showed varying degrees of upregulation or downregulation. Furthermore, overexpression of *GmMLP9* significantly enhanced salt stress tolerance in both yeast and soybean plants. The soybean composite plants with *GmMLP9*-overexpressing hairy roots exhibited a lower Na^+^/K^+^ ratio and lower MDA content, but higher proline content.

**Discussion:**

This study provides a theoretical foundation for further exploration of the *MLP* gene family’s role in soybean adaptation to salt stress and offers valuable insights for identifying candidate salt-tolerance genes and advancing molecular breeding efforts

## 1 Introduction

Major latex proteins (MLPs) are a category of plant-specific, low-molecular-weight proteins first identified in the latex of the opium poppy (Nessler et al., 1985). The term ‘Major latex protein’ is derived from their high abundance in plant latex; however, subsequent studies have demonstrated that MLPs are also present in various dicotyledonous plants. Based on sequence homology, MLPs are classified into three main groups: MLP, Bet v 1, and pathogenesis-related protein 10 (PR-10). Although most current research has focused on Bet v 1 and PR-10, studies specifically addressing MLPs remain relatively limited (Fujita and Inui, 2021; Aglas et al., 2020). Recently, MLPs have attracted increasing attention due to their diverse biological functions, which appear to be broader than those of the other two related proteins.

MLP proteins are characterized by a well-conserved helix-grip fold, which consists of three to four α-helices (α1, α2, α3) and seven β-strands that encircle α3. Collectively, these components form a Y-shaped hydrophobic pocket within the protein (Lytle et al., 2009). Studies have shown that this hydrophobic region can bind not only endogenous plant hormones, such as abscisic acid and various hydrophobic ligands, but also more complex amphipathic molecules (Mattila and Renkonen, 2009; Aglas et al., 2020). A heteronuclear single quantum coherence (HSQC) titration experiment conducted on *Arabidopsis thaliana* demonstrated that the AT1G24000 protein can interact with the hydrophobic hormone progesterone, which is not typically found in plants, thereby highlighting the functional stability of this hydrophobic pocket (Lytle et al., 2009). Although the primary sequences of MLP family proteins differ considerably, their tertiary structures and functional sites remain largely conserved. MLP proteins contain a conserved glycine-rich loop characterized by the common sequence motif GXGGXG, which resembles the RNase active site found in PR-10 proteins (Biesiadka et al., 2002; Fernandes et al., 2013). Interestingly, despite the presence of multiple glycine-rich loops, there is no evidence to suggest that MLPs exhibit RNase activity (Fujita and Inui, 2021).

The *MLP* gene family has been extensively identified across various plant species, including *Arabidopsis thaliana* (Lytle et al., 2009), tomato (Sun et al., 2023), peanut (Li et al., 2023), cucumber (Kang et al., 2023), apple (Yuan et al., 2020), poplar (Sun et al., 2024), grape (Zhang et al., 2018), and rose (Chen et al., 2024). The variation in the number of *MLP* family members among these species indicates that this gene family has undergone different degrees of expansion and specialization throughout plant evolution. Analysis of expression profiles reveals that *MLP* genes in different species exhibit distinct expression patterns influenced by developmental stages, tissue types, hormonal treatments, and various stress factors, including extreme temperatures, salinity, drought, waterlogging, and pathogen attacks. Notably, several *MLP* genes in tomato, peanut, poplar, and grape respond to abiotic stressors (Zhang et al., 2018; Li et al., 2023; Sun et al., 2023, 2024), whereas certain *MLP* genes in cucumber, apple, and rose are associated with pathogen responses (Yuan et al., 2020; Kang et al., 2023; Chen et al., 2024). Currently, most research on plant *MLP* genes has focused on their identification and expression, while their specific functions and mechanisms remain largely unexplored.

Studies have demonstrated that MLPs can bind to small molecule ligands and play significant roles in hormone signaling, abiotic stress responses, and pathogen defense. In opium poppy, MLP/PR10 proteins interact with benzylisoquinoline alkaloids (BIAs), influencing their metabolism and accumulation (Ozber et al., 2022). In *Arabidopsis*, the AtMLP43 protein has been identified as interacting with SnRK2.6 and ABF1, with plants overexpressing it exhibiting enhanced drought resistance (Wang et al., 2016). In cucumber, CsMLP1 is known to enhance resistance to *Phytophthora melonis*, whereas CsMLP5 appears to exert an opposing effect on disease resistance (Kang et al., 2023). The heterologous expression of *GhMLP* from cotton has been shown to increase salt stress tolerance and boost flavonoid levels in *Arabidopsis* (Chen and Dai, 2010). Additionally, GhMLP28 enhances the interaction of GhERF6 with the GCC-box, thereby improving cotton’s defense against *Verticillium dahliae* (Yang et al., 2015). In apple, the overexpression of *MdMLP423* has been linked to decreased resistance to fungal infections (He et al., 2020), highlighting the functional diversity within the MLP family. Furthermore, the promoter region of *NbMLP28* contains hormones and abiotic stress-responsive cis-elements, including MeJA and drought-responsive elements, and NbMLP28 enhances resistance to *Potato virus Y* (PVY), potentially through the jasmonic acid signaling pathway signaling (Song et al., 2020). Moreover, overexpressing *NtMLP423* has been shown to significantly enhance cold tolerance in transgenic tobacco (Liu et al., 2023). Research has also revealed that *MLP* promoters in poplar and grape contain cis-acting elements associated with salt stress and low temperatures, suggesting a role for this gene family in regulating stress signals (Zhang et al., 2018; Sun et al., 2024). In soybean, the *MLP* homolog *Msg*, one of the earliest identified, was cloned from developing pods. Promoter truncation assays revealed that *Msg* is highly expressed in pods, with its promoter activity primarily controlled by distal regulatory regions rather than the proximal 650 bp TATA-containing segment (Strömvik et al., 1999; Vodkin et al., 2003). However, previous investigations have predominantly focused on this single soybean *MLP* gene.

The soybean (*Glycine max*) is an essential crop worldwide, valued for its applications in oil, food, and animal feed. However, its growth and productivity are significantly impacted by salt stress. Research indicates that salt stress is a primary abiotic factor that hinders both the yield and quality of soybeans, triggering extensive transcriptome reprogramming in the plant, which significantly influences its growth and development (Essa, 2002; Liu et al., 2019). Given that salinity substantially reduces soybean yield and seed quality and causes severe economic losses worldwide, identifying genes that confer salt tolerance is essential for stabilizing soybean production and improving the utilization of salt-affected farmland. Despite the importance of soybeans as a legume, studies focusing on *MLP* genes within this species remain limited, particularly regarding comprehensive genome-wide analyses. This research conducted a thorough identification and analysis of the *MLP* gene family in soybeans, examining phylogenetic relationships, gene structure, and promoter cis-acting elements, thereby illuminating the evolutionary characteristics and regulatory functions of this gene family. Furthermore, the study investigated the varying expression patterns of *MLP* genes in response to salt stress and ABA treatment, confirming the role of the *GmMLP9* gene in the soybean’s response to salt stress.

## 2 Materials and methods

### 2.1 Genome-wide identification and phylogenetic analysis of GmMLP family genes

Whole-genome sequences and genome annotation files were obtained from the Phytozome plant genome database (https://phytozome-next.jgi.doe.gov). MLP family protein sequences from *Arabidopsis*, tomato and peanut were retrieved from the same database and used as reference sequences. An HMM profile was constructed using HMMER3.0 (http://hmmer.org) from the identified MLP proteins and employed to search the soybean proteome for candidate MLP family members. Concurrently, all soybean protein sequences were searched against the reference MLP sequences using BLASTP (NCBI BLAST v2.10.1+) with an E-value cutoff of 1e^-5^ (Camacho et al., 2009). The sequences recovered from these searches were combined as candidate GmMLP proteins. Domain annotation of the candidate sequences was then performed using PfamScan v1.6 and the Pfam-A database v33.1 (Mistry et al., 2021). Candidates containing the Bet_v_1 domain (PF00407) were retained as the final GmMLP sequences. To relate the GmMLP proteins identified in this study to previously reported soybean MLP proteins, protein sequences corresponding to available NCBI accessions were compared against the 41 GmMLP proteins using BLASTP. Matches were evaluated based on sequence identity, query coverage, alignment length, E-value, mismatches, and gaps. MLP protein sequences from soybean, *Arabidopsis*, tomato and peanut were aligned using MUSCLE5 (Edgar, 2022). A phylogenetic tree was constructed in MEGA12 using the neighbor-joining method with 1000 bootstrap replicates (Kumar et al., 2024). The resulting tree was annotated and visualized using iTOL v6 (https://itol.embl.de/).

### 2.2 Physicochemical properties and subcellular localization prediction of GmMLP family proteins

The physicochemical properties of soybean MLP proteins were calculated using ExPASy ProtParam (https://web.expasy.org/protparam/). The parameters analyzedanalyze meter included protein length, molecular mass, theoretical isoelectric point, instability index and hydropathicity. Subcellular localization was predicted using WoLF PSORT (https://wolfpsort.hgc.jp).

### 2.3 Multiple sequence alignment, gene structure and conserved motif analysis of the *GmMLP* family

Multiple sequence alignment and visualization of soybean MLP protein sequences were conducted using MUSCLE5 and TBtools. Gene structure information for *GmMLPs* was extracted from the soybean GFF3 annotation file. Conserved motifs within the *GmMLP* family were identified using the MEME Suite (https://meme-suite.org/meme/tools/meme), with the motif search parameter set to 15. Gene structures and conserved motifs were visualized using TBtools (Chen et al., 2023).

### 2.4 Chromosomal localization, synteny, and evolutionary selection pressure analysis

The genomic locations of *GmMLP* genes were obtained from the soybean GFF3 annotation file and visualized on chromosomes using MG2C v2.1 (http://mg2c.iask.in/mg2c_v2.1/). Syntenic relationships among soybean *MLP* family members, as well as between the soybean and *Arabidopsis*, tomato, and peanut, were analyzed using MCScanX (Wang et al., 2012). The synteny results were visualized using TBtools. Additionally, the Ka and Ks values for duplicated gene pairs were calculated using KaKs_Calculator v2.0 (Wang D. et al., 2010).

### 2.5 Prediction and analysis of cis-acting elements in the *GmMLP* genes

Promoter sequences, defined as the 2,000 bp upstream regions of soybean *MLP* genes, were extracted for cis-element analysis. Cis-acting elements in the *GmMLP* promoters were identified using the PlantCARE database (Lescot et al., 2002). The 30 most frequently detected cis-acting elements were selected for downstream visualization, excluding the CAAT-box and TATA-box elements. Heatmaps and stacked bar charts were generated in R to summarize the distribution of these elements.

### 2.6 Expression profiles of *GmMLP* genes in different tissues and salt-stressed roots

Gene expression data from various soybean tissues were sourced from the Soybean Expression Atlas v2 (Almeida-Silva et al., 2023). The expression of *GmMLP* was analyzed in roots, leaves, cotyledons, flowers, pods, nodules, and seeds. TPM values were transformed to log2 (TPM + 1) values. For tissues with multiple RNA-seq samples, the mean expression value was calculated and used for heatmap visualization.

For the salt-stress transcriptome analysis, uniformly grown seedlings of the soybean cultivar Qihuang 34 (QH34) were treated with 150 mM NaCl (Huang et al., 2021; Hu et al., 2022). Root samples were collected at 1, 3, 6 and 12 h after treatment for transcriptome analysis. Control samples were collected from plants grown in a standard nutrient solution. Total RNA was isolated using the FastPure Universal Plant Total RNA Isolation Kit (Vazyme, Nanjing, China; Cat. No. RC411), resulting in high-quality RNA suitable for subsequent analyses. RNA sequencing was performed on the Illumina HiSeq 4000 system at Novogene Bioinformatics Technology (Beijing, China), where the data analysis was also conducted by Novogene. Clean RNA-seq reads were aligned to the soybean reference genome (Williams 82.a4.v1) using TopHat (version 2.0.12). Differentially expressed genes under salt stress were identified using thresholds of an adjusted *P* value <0.05 and an absolute log_2_ fold change ≥2 with DESeq2 (Wang L. et al., 2010). Expression values for *GmMLP* family genes were extracted from these data and visualized as a heatmap.

### 2.7 Identification of *GmMLPs* genes in response to the NaCl and ABA treatment

Healthy Qihuang 34 (QH34) soybean seeds with uniform size and appearance were selected and germinated in quartz sand for 3 d. The seedlings were then transferred to a half-strength modified Hoagland nutrient solution for 3 d of acclimation. Subsequently, the seedlings were grown in a modified Hoagland nutrient solution supplemented with either 150 mM NaCl (Hu et al., 2022) or 100 μM ABA (Li et al., 2019; Liu et al., 2026). Root samples were collected at 0, 3, 6, 9 and 12 h after treatment, immediately frozen in liquid nitrogen, and stored at -80 °C until RNA extraction and RT-qPCR analysis.

### 2.8 qRT-PCR analysis

Total RNA was extracted from the collected samples using the RNAiso Plus reagent (Takara, Japan) according to the manufacturer’s instructions. First-strand cDNA was synthesized from the extracted total RNA and used as the template for RT-qPCR analysis. RT-qPCR was used to measure the expression levels of 19 selected *GmMLP* genes under NaCl and ABA treatments. Each treatment consisted of three biological replicates, with each biological replicate analyzed using three technical replicates. Reactions were performed using the ChamQ Universal SYBR qPCR Master Mix (Vazyme, China) on a CFX Duet Real-Time PCR System (Bio-Rad, USA). *GmActin* (*Glyma.18G290800*) was utilized as the internal reference gene for normalization (Ma et al., 2013; Yim et al., 2015). Relative expression levels were calculated using the 2^−ΔΔCt^ method. The RT-qPCR primers are listed in **Supplementary Table 1**. Data are presented as the mean ± standard deviation.

### 2.9 Functional validation of *GmMLP9* heterologous expression in yeast

*GmMLP9* was amplified using the primers *GmMLP9*-F2 and *GmMLP9*-R2 (**Supplementary Table 1**). The amplified fragment was subsequently cloned into the pYES2-NTB vector via homologous recombination. The recombinant plasmid was then introduced into the yeast strain INVSC1 using the PEG/LiAc method. Positive clones carrying pYES2-NTB-*GmMLP9* or the empty pYES2-NTB vector were resuspended in 2 mL of sterile water and serially diluted to concentrations of 10^0^, 10^−1^ and 10^−2^. The diluted suspensions were spotted onto SG-Ura plates containing 0, 0.5, 1.0, or 1.2 M NaCl. The plates were incubated at 30 °C for one week before observation and imaging.

For the liquid growth assay, yeast cells carrying pYES2-NTB or pYES2-NTB-*GmMLP9* were cultured in SG-Ura medium or SG-Ura medium supplemented with 1.0 M NaCl. OD600 was measured at 0, 6, 12, 24, 36, 48, and 72 h. Three independent biological replicates were performed, and data are presented as mean ± SD. This OD600-based growth-curve strategy follows commonly used yeast heterologous expression assays for plant salt-stress-related genes (Guo et al., 2025).

### 2.10 The induction of soybean hair roots and phenotypic analysis under salt stress

For soybean hairy-root transformation, *GmMLP9* was amplified using the primers *GmMLP9*-F3 and *GmMLP9*-R3 (**Supplementary Table 1**) and inserted into the pCAMBIA3301 expression vector via homologous recombination to generate the 35S-*GmMLP9* overexpression construct. The construct was then introduced into *Agrobacterium rhizogenes* K599 using the freeze-thaw method. Soybean hypocotyls were transformed with *A. rhizogenes* carrying the 35S-*GmMLP9*, and the empty pCAMBIA3301 vector was used as the control. Following the emergence of hairy roots, the plants were transferred to sterilized vermiculite and allowed to acclimate for 3 days before being subjected to 150 mM NaCl treatment. After 7 days of treatment, salt-stress phenotypes were photographed, and samples were collected for subsequent analyses. The contents of Na^+^ and K^+^ were measured using a flame spectrophotometer (Sherwood, M410) following the method described for salt-treated plant tissues (Munns et al., 2010). The contents of malondialdehyde (MDA) and proline were assessed using the MDA Quantification Assay Kit (Coolaber, Beijing, China) and the Proline Content Assay Kit (Solarbio, Beijing, China), respectively. All experiments were conducted with three biological replicates.

### 2.11 Prediction of the GmMLP9 protein–protein association network

To predict potential GmMLP9-associated proteins, the GmMLP9 protein accession (UniProt: I1KMV3) was queried in the STRING database using Glycine max as the reference organism (Szklarczyk et al., 2023). The interaction table was exported from STRING and subsequently employed for network visualization in R. The edge width represented the STRING combined confidence score, while the node size indicated the degree of each node.

### 2.12 Statistical analyses

All experiments were conducted with three biological replicates. Statistical significance among groups was assessed using a one-way analysis of variance (ANOVA), followed by Tukey’s multiple-comparison test. Different letters above the bars indicate significant differences at *P* < 0.05.

## 3 Results

### 3.1 Genome-wide identification of MLP family genes in soybean

In this study, we identified 41 members of the soybean MLP family, designated from GmMLP1 to GmMLP41. The proteins encoded by these *GmMLP* genes varied in length from 125 amino acids for GmMLP27 to 319 amino acids for GmMLP28. The molecular weights (MW) of these 41 GmMLP proteins ranged from 13824.68 Da for GmMLP27 to 34985.06 Da for GmMLP28. The predicted isoelectric points (PI) varied from 4.57 for GmMLP6 to 8.25 for GmMLP33. Notably, all soybean MLP proteins exhibited grand averages of hydropathicity (GRAVY) values exceeding 0. Predictions regarding subcellular localization indicated that 35 of these members are localized in the cytoplasm, 3 in the chloroplast, 1 in the cytoskeleton, while the remaining 2 are secreted into the extracellular space (**Table 1**).

**Table 1:** List of major latex protein family genes from soybean.

Gene Name	Gene ID	CDS	Chromosome			Stand	Protein				Subcellular
		Length	No.	Start	End		length	MW(Da)	Theoretical pI	Grand average of hydropathicity	Localization
*GmMLP1*	*Glyma.01G034000*	462	Gm01	3552884	3554937	–	153	17039.12	4.99	0.432	cyto
*GmMLP2*	*Glyma.02G032300*	462	Gm02	2930653	2933087	+	153	17104.24	5.1	0.424	cyto
*GmMLP3*	*Glyma.02G192400*	426	Gm02	38201155	38202114	+	141	16153.48	6.04	0.372	cyto
*GmMLP4*	*Glyma.06G249800*	465	Gm06	41614674	41616113	+	154	17310.63	4.79	0.139	cyto
*GmMLP5*	*Glyma.07G219700*	381	Gm07	39669578	39670336	+	126	14496.49	5.62	0.533	cyto
*GmMLP6*	*Glyma.07G243500*	498	Gm07	42651953	42653004	–	165	17599.7	4.57	0.266	cyto
*GmMLP7*	*Glyma.07G243600*	477	Gm07	42664436	42665367	+	158	16760.81	4.73	0.246	cyto
*GmMLP8*	*Glyma.07G243651*	477	Gm07	42656771	42657698	+	158	16771.81	4.69	0.272	cyto
*GmMLP9*	*Glyma.07G243900*	483	Gm07	42675788	42676798	+	160	17979.31	4.61	0.351	chlo
*GmMLP10*	*Glyma.08G230100*	459	Gm08	18897684	18899181	–	152	17619.71	5.38	0.572	cyto
*GmMLP11*	*Glyma.08G230400*	459	Gm08	18920530	18921624	+	152	17352.61	6.02	0.436	cyto
*GmMLP12*	*Glyma.08G230500*	462	Gm08	18936036	18937630	–	153	17761.88	5.96	0.622	cyto
*GmMLP13*	*Glyma.09G040400*	477	Gm09	3341270	3342584	+	158	16886.05	4.88	0.277	cyto
*GmMLP14*	*Glyma.09G040500*	474	Gm09	3346524	3347860	+	157	17139.55	5.34	0.065	cyto
*GmMLP15*	*Glyma.09G040600*	477	Gm09	3362067	3364617	+	158	16532.54	4.68	0.108	cyto
*GmMLP16*	*Glyma.09G040800*	960	Gm09	3373071	3375895	+	319	34813.67	4.91	0.232	cyto
*GmMLP17*	*Glyma.09G102400*	468	Gm09	18243576	18245776	–	155	17391.85	6.06	0.356	cyto
*GmMLP18*	*Glyma.09G118300*	462	Gm09	29224583	29226201	–	153	17333.49	5.63	0.525	cysk
*GmMLP19*	*Glyma.10G084400*	462	Gm10	10261818	10264180	–	153	17534.9	5.35	0.442	cyto
*GmMLP20*	*Glyma.12G017100*	486	Gm12	1201719	1203036	–	161	17705.19	4.81	0.214	cyto
*GmMLP21*	*Glyma.13G030200*	546	Gm13	8671090	8672835	+	181	20797.85	6.03	0.181	extr
*GmMLP22*	*Glyma.15G145600*	477	Gm15	11983629	11985085	+	158	16861.04	4.9	0.237	cyto
*GmMLP23*	*Glyma.15G145700*	474	Gm15	11987261	11989250	+	157	17175.63	5.59	0.05	cyto
*GmMLP24*	*Glyma.15G145800*	474	Gm15	12005924	12006782	+	157	17152.57	5.51	0.066	cyto
*GmMLP25*	*Glyma.15G145900*	465	Gm15	12011076	12013028	+	154	16141.31	5.17	0.036	cyto
*GmMLP26*	*Glyma.15G146000*	585	Gm15	12014317	12015314	+	194	21513.8	5.07	0.009	extr
*GmMLP27*	*Glyma.15G146100*	378	Gm15	12023557	12024946	+	125	13824.68	4.71	0.168	cyto
*GmMLP28*	*Glyma.15G146400*	960	Gm15	12038707	12041883	+	319	34985.06	5.36	0.251	cyto
*GmMLP29*	*Glyma.15G146500*	483	Gm15	12042387	12043684	+	160	17716.04	4.73	0.266	cyto
*GmMLP30*	*Glyma.15G146600*	483	Gm15	12047365	12048666	+	160	17901.43	4.79	0.25	cyto
*GmMLP31*	*Glyma.15G217100*	459	Gm15	36759935	36761095	–	152	17562.75	6.02	0.466	cyto
*GmMLP32*	*Glyma.15G218900*	459	Gm15	38397161	38398331	–	152	17623.88	6.24	0.574	cyto
*GmMLP33*	*Glyma.15G274200*	468	Gm15	53175983	53178028	–	155	17637.45	8.25	0.339	chlo
*GmMLP34*	*Glyma.15G274300*	468	Gm15	53182550	53184192	–	155	17678.12	6.05	0.445	cyto
*GmMLP35*	*Glyma.16G079800*	456	Gm16	8382841	8384176	+	151	17187.37	5.32	0.466	cyto
*GmMLP36*	*Glyma.17G030000*	483	Gm17	2202210	2203173	–	160	17846.21	4.65	0.292	chlo
*GmMLP37*	*Glyma.17G030100*	474	Gm17	2209060	2210221	–	157	17112.21	4.98	0.317	cyto
*GmMLP38*	*Glyma.17G030200*	477	Gm17	2214439	2215414	–	158	16756.91	4.93	0.187	cyto
*GmMLP39*	*Glyma.17G030300*	477	Gm17	2217580	2218493	+	158	16728.85	4.92	0.189	cyto
*GmMLP40*	*Glyma.17G030400*	477	Gm17	2220873	2221731	–	158	16711.76	4.8	0.246	cyto
*GmMLP41*	*Glyma.20G017900*	462	Gm20	1830851	1831818	+	153	17721.19	6.25	0.603	cyto

### 3.2 Phylogenetic classification of MLP proteins

**Figure 1:**
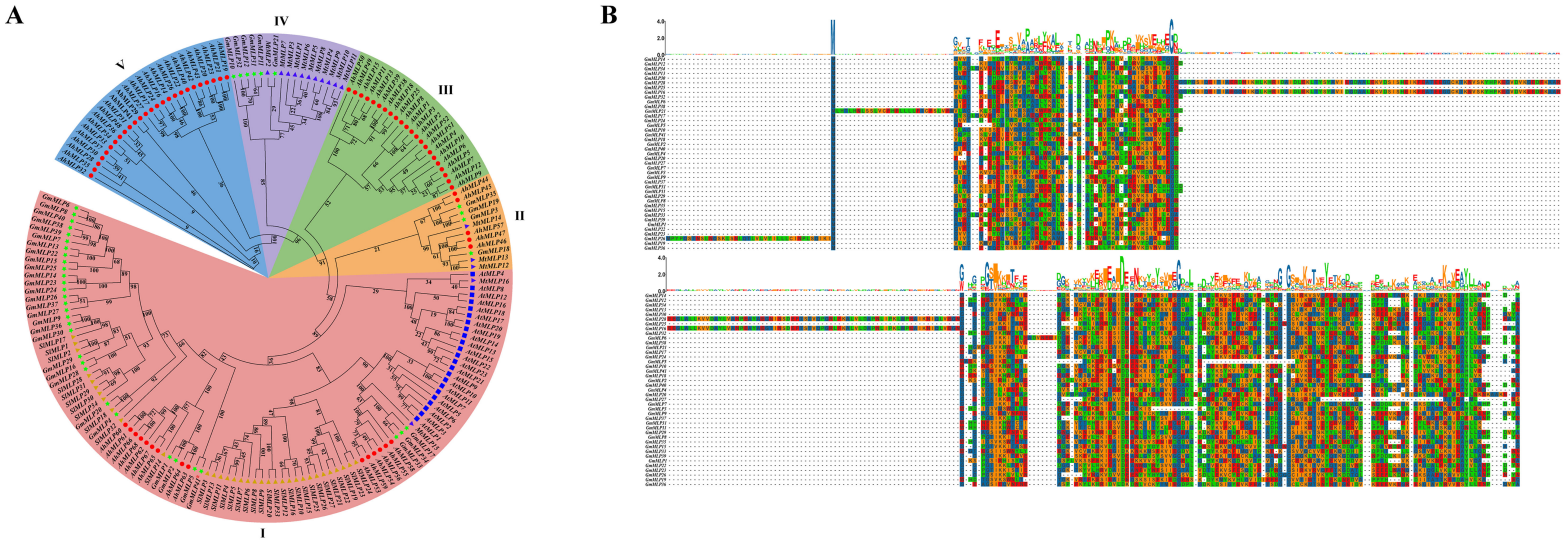
Phylogenetic analysis and multiple sequence alignment of soybean *GmMLP* family members. **(A)** Phylogenetic tree of the GmMLP proteins and reported MLP proteins from *Arabidopsis thaliana*, *Arachis hypogaea*, *Medicago truncatula*, and *Solanum lycopersicum*. The tree was constructed based on full-length protein sequences using Neighbor-joining with 1000 bootstrap replicates. **(B)** Multiple sequence alignment of the GmMLP proteins. Conserved amino acid residues highlighted according to the alignment.

To systematically elucidate the evolutionary relationships among members of the soybean MLP family, we constructed a phylogenetic tree utilizing 41 identified soybean MLP protein sequences along with previously documented MLP protein sequences from species such as *Arabidopsis*, *Medicago truncatula*, *Arachis hypogaea*, and *Solanum lycopersicum* (**Figure 1A**; **Supplementary Table 2**). The results indicated that all MLP proteins could be categorized into five distinct groups (I–V), with the majority of soybean MLP proteins located in subgroups I, II, and IV. Group I, the most populous, encompasses MLP proteins from various species, indicating significant gene expansion. Although the other four groups contained fewer members, each branch exhibited several well-supported subclades, reflecting closer genetic ties among those members.

### 3.3 Multiple sequence alignment

Multiple sequence alignments revealed that most GmMLP proteins shared a conserved central region, whereas the N-terminal regions were more variable (**Figure 1B**). Notably, GmMLP16 and GmMLP28 were significantly longer than most other GmMLP members and contained a unique insertion region specific to these two proteins in the alignment, where the corresponding positions in most other GmMLP proteins were represented by gaps. This sequence characteristic indicated structural variation among GmMLP members.

### 3.4 Conserved motif and gene structure analysis

**Figure 2:**
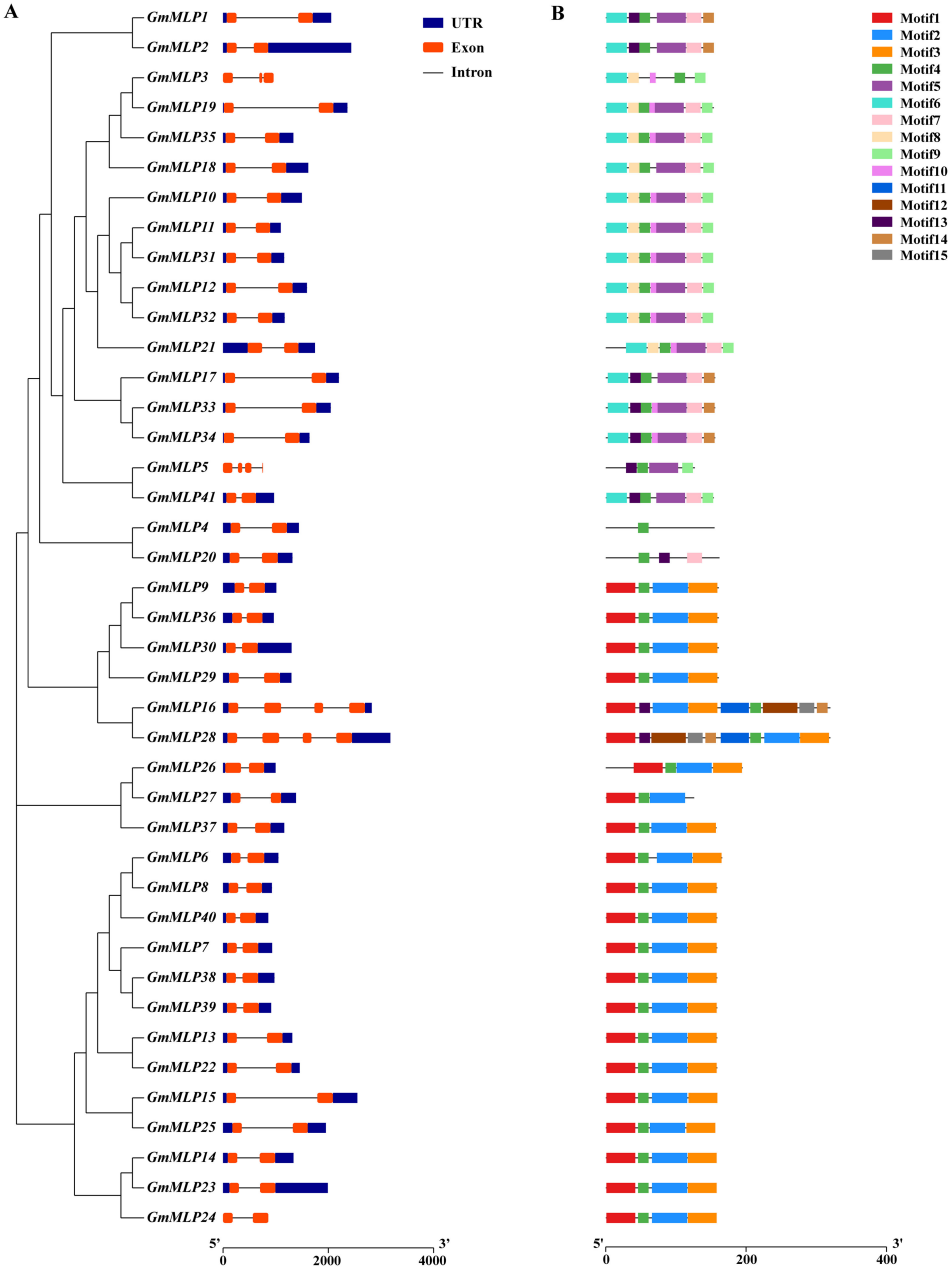
Gene structure and conserved motif analysis of *GmMLP* family members. **(A)** Structures of *GmMLP* genes based on their genomic and coding sequences. Exons, introns, and UTR are shown according to the schematic legend. **(B)** Conserved motifs of GmMLP proteins predicted using MEME. Different colored boxes represent different MEME-predicted conserved motifs, while the gray lines indicate the length of the protein sequences.

Analysis of the exon-intron structures of the soybean *MLP* gene family revealed considerable variations in both the number and distribution of exons and introns among different *GmMLP* genes (**Figure 2A**). Notably, members sharing the same terminal branch in the phylogenetic tree tend to exhibit similar gene structures. The majority of *GmMLP* genes display a conserved structure, typically comprising two exons and one intron. However, certain members, such as *GmMLP16* and *GmMLP28*, are characterized by more complex structures that include four exons. Furthermore, three members (*GmMLP3*, *GmMLP5*, and *GmMLP24*) lack discernible UTRs, highlighting the variability in the lengths and structures of non-coding region among these genes. MEME analysis of conserved motifs revealed differences in both the quantity and arrangement of these motifs across various GmMLP proteins, whereas those within the same phylogenetic group displayed a high degree of consistency in motif composition and order (**Figure 2B**; **Supplementary Table 3**). For instance, GmMLP6, GmMLP8, GmMLP40, GmMLP7, and GmMLP38 shared a similar arrangement of Motif 1, 4, 2, and 3, suggesting structural conservation and a close evolutionary relationship among these proteins. Notably, Motif4 appears in nearly all GmMLP members, indicating that it may represent a fundamental conserved element of the GmMLP proteins family or be linked to essential functions. GmMLP20 and GmMLP27 exhibit fewer motifs, while GmMLP4 contains only Motif4, suggesting that some genes may have undergone structural simplification and functional specialization. In contrast, GmMLP16 and GmMLP28 display a wider variety of motifs and more complex structures, hinting at functional differentiation over time. Overall, the *GmMLP* gene family exhibits both conservation and differentiation in gene structure and motif composition. Members of the same evolutionary clade typically demonstrate similar structures and motif arrangements, thereby reinforcing the grouping outcomes of the phylogenetic tree.

### 3.5 Chromosomal distribution, duplication patterns, and synteny relationships of *MLP* genes

**Figure 3:**
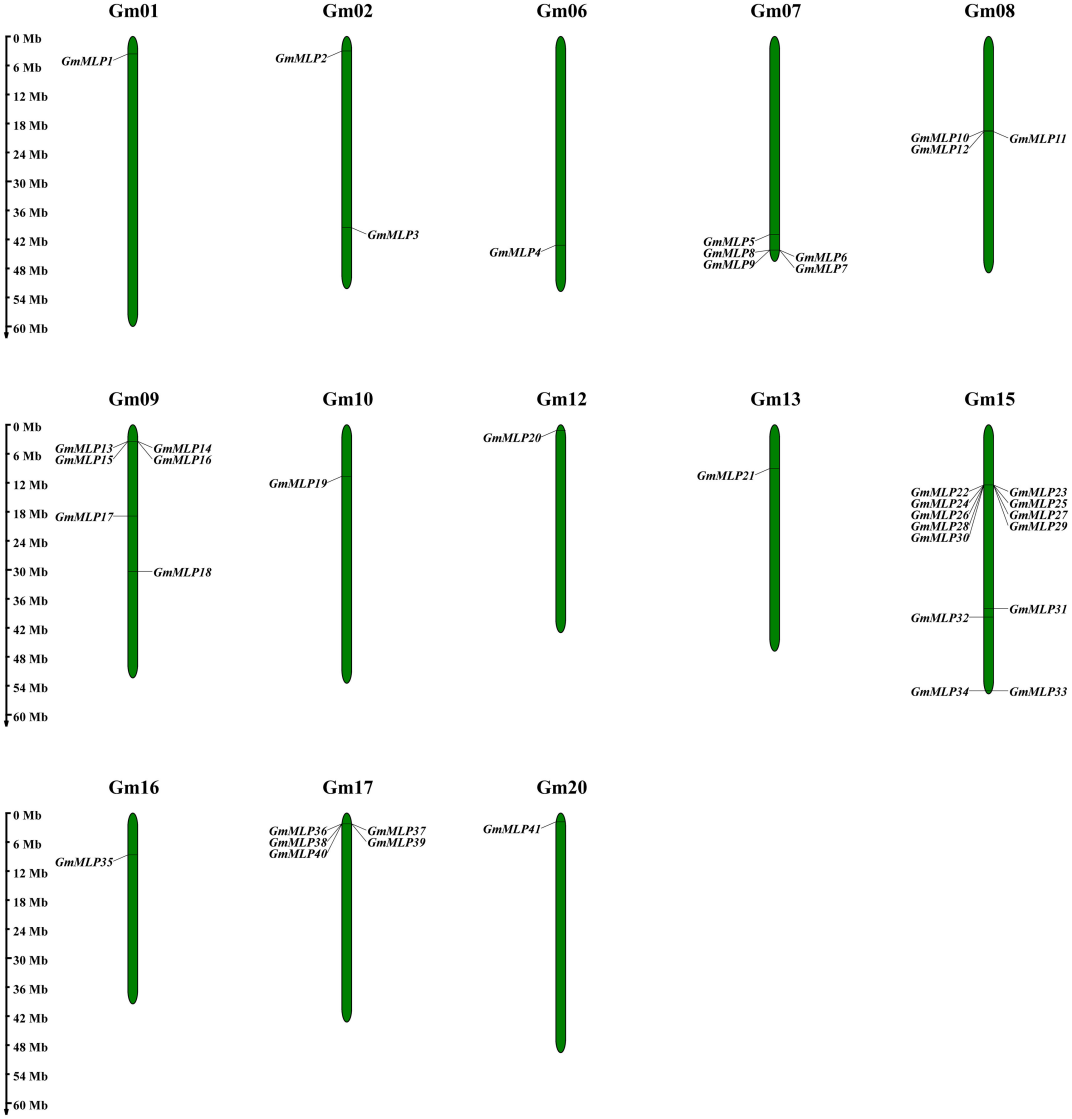
Chromosomal distribution of *GmMLP* genes. The chromosomal locations of *GmMLP* genes in soybean. Green bars represent the 20 soybean chromosomes. The chromosome numbers are presented at the tops of the green bars. Scale bar on the left represents the lengths of each chromosome (Mb).

The chromosomal positions of *GmMLP* genes were obtained from the GFF3 annotation file of the *Glycine max* Williams 82 genome assembly (Wm82.a4.v1), which was downloaded from Phytozome (Goodstein et al., 2012; Valliyodan et al., 2019). Our results indicated an uneven distribution of these genes, with their presence observed on only 13 chromosomes. Specifically, chromosomes Chr07, Chr09, Chr15, and Chr17 exhibited a higher density of *MLP* genes, while the remaining chromosomes displayed a lower frequency of these genes (**Figure 3**).

**Figure 4:**
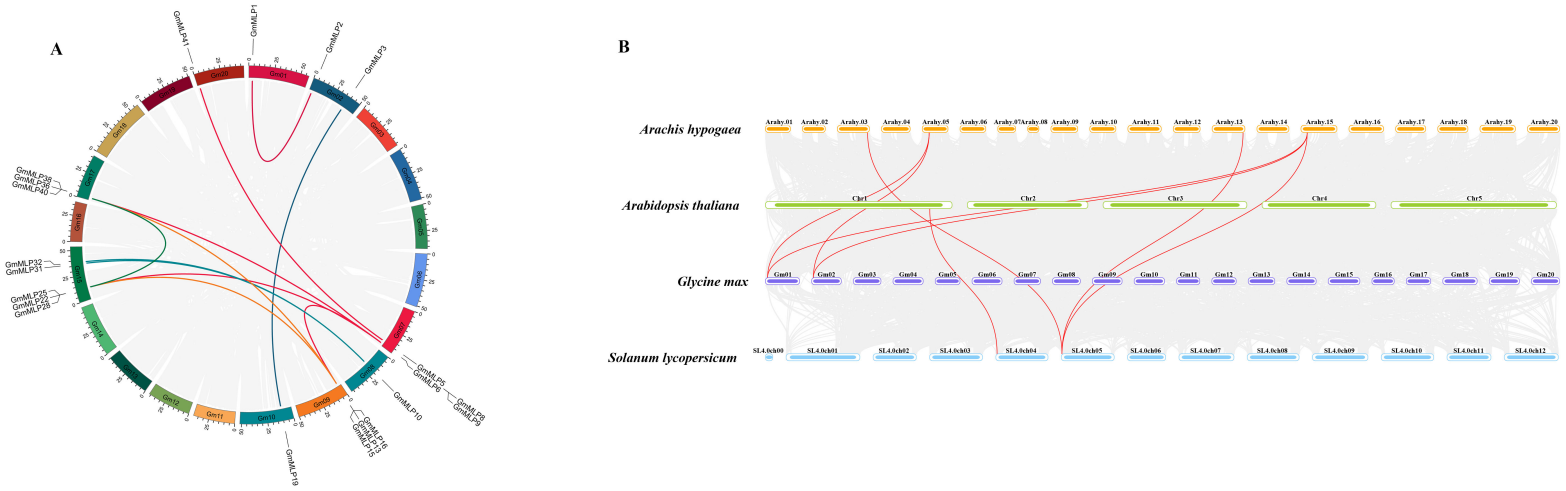
Collinearity analysis of *GmMLP* genes within soybean and between soybean and other plant species. **(A)** Intraspecific collinearity analysis of *GmMLP* genes in the soybean genome. Collinear or duplicated *GmMLP* gene pairs are connected by lines. **(B)** Interspecific collinearity analysis of *MLP* homologous genes between *Glycine max* and *Arabidopsis thaliana*, *Arachis hypogaea*, and *Solanum lycopersicum*. Lines indicate syntenic homologous gene pairs between soybean and the corresponding species.

To investigate the replication and distribution patterns of the *MLP* gene family within the soybean genome, an intra-species synteny analysis was conducted on the 41 *GmMLP* genes (**Figure 4A**). The findings revealed 16 pairs of syntenic relationships among these genes, distributed across various chromosomes, with notable clusters located in the regions of Chr7-Chr9, Chr15, and Chr17. Analysis of the physical locations of these genes on the chromosomes suggested that the syntenic relationships among *GmMLP* genes resulted from segmental duplication events. Furthermore, an assessment of the selection pressure on the duplicated gene pairs indicated that, apart from *GmMLP24*/*GmMLP34* and *GmMLP19*/*GmMLP7*, all other pairs exhibited Ka/Ks ratios below 1.

We also explore the evolutionary links of the *MLP* gene family across various species by analyzing the syntenic relationships of related genes in *Arabidopsis thaliana*, *Arachis hypogaea* and *Solanum lycopersicum* (**Figure 4B**). Our finding revealed that the number of syntenic gene pairs varied among these species, with overall syntenic connections being relatively sparse. Specifically, we identified four syntenic pairs between soybean and peanut, three between tomato and peanut, and only one between tomato and *Arabidopsis*. These results indicated a certain level of conservation among *MLP* gene family members across species. The most significant syntenic relationship was observed between soybean and peanut, suggesting a higher degree of evolutionary conservation within leguminous plants. Furthermore, some collinearity was detected between tomato and peanut, implying that this gene family has retained specific homologous genes across various dicotyledonous groups. In contrast, only one collinear gene pair was identified between tomato and *Arabidopsis*, highlighting a reduced level of conservation between these two species, which may be attributed to different rates of gene loss, duplication, or functional divergence.

### 3.6 Cis-acting regulatory element analysis

**Figure 5:**
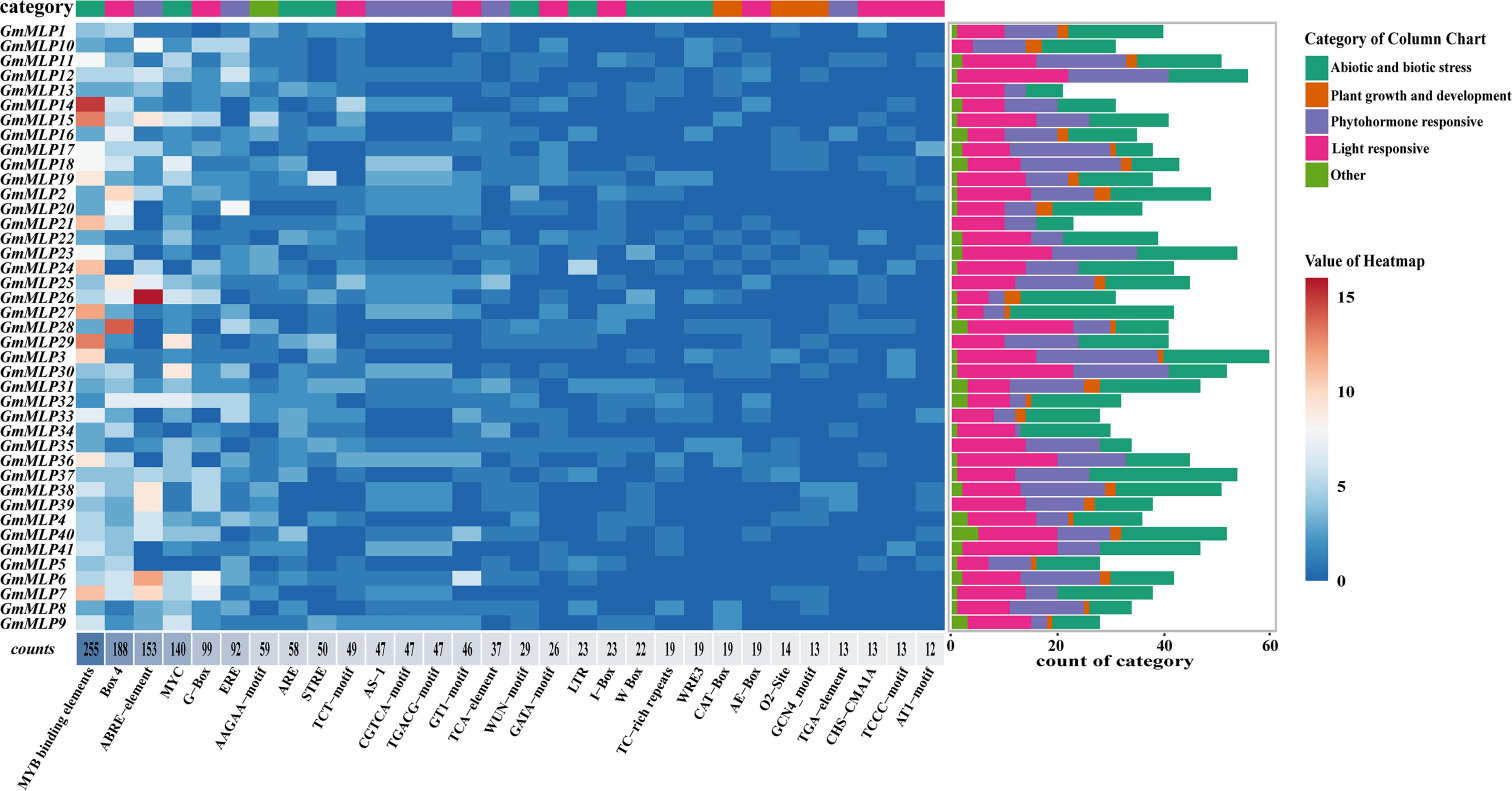
Predicted cis-acting regulatory elements in the promoter regions of *GmMLP* genes. Distribution of predicted cis-acting regulatory elements in the promoter regions of *GmMLP* genes. Promoter sequences upstream of the *GmMLP* coding regions were analyzed using PlantCARE. Different colors or symbols indicate different categories of cis-acting elements, including hormone-responsive, stress-responsive, light-responsive, growth or development-related elements and other elements.

To further investigate the transcriptional regulatory mechanisms of *GmMLP* genes, we conducted a comprehensive analysis of the *cis*-acting elements present in 41 soybean *MLP* genes. Our results revealed that the promoter regions of all *GmMLP* genes are characterized by a diverse array of cis-acting elements, with notable variations in both type and quantity across different gene members (**Figure 5**). Key transcriptional components, such as the TATA box and CAAT box, were identified in each *GmMLP* promoter, suggesting that these genes possess essential structures for transcription initiation. The identified cis-acting elements can be categorized into five functional groups: stress-responsive elements, growth and development-related elements, hormone-responsive elements, light-responsive elements, and additional elements associated with basic transcription functions.

Additionally, we identified a variety of stress-responsive cis-acting elements, including binding sites for MYB and MYC transcription factors, the stress-responsive element (STRE), the wound-responsive WUN-motif, the anaerobic-responsive element (ARE), the WRKY transcription factor binding site (W-Box), and the low-temperature-responsive element (LTR). Notably, elements associated with MYB were the most prevalent, with 255 occurrences, representing 15.51% of the overall count. Furthermore, we identified several regulatory elements linked to plant hormones, including the abscisic acid-responsive element (ABRE), the ethylene-responsive element (ERE), the CGTCA/TGACG motif responsive to methyl jasmonate (MeJA) and various hormone-responsive and stress defense elements such as as-1, the salicylic acid-responsive TCA-element, and the auxin-responsive TGA-element. Among these, ABRE was the most frequently observed, appearing 153 times and accounting for 9.31% of the total, placing it just behind the MYB-related elements and the light-responsive Box4 in terms of quantity (**Figure 5**).

### 3.7 Expression profiles of *MLP* genes in different tissues and salt stress transcriptome

**Figure 6:**
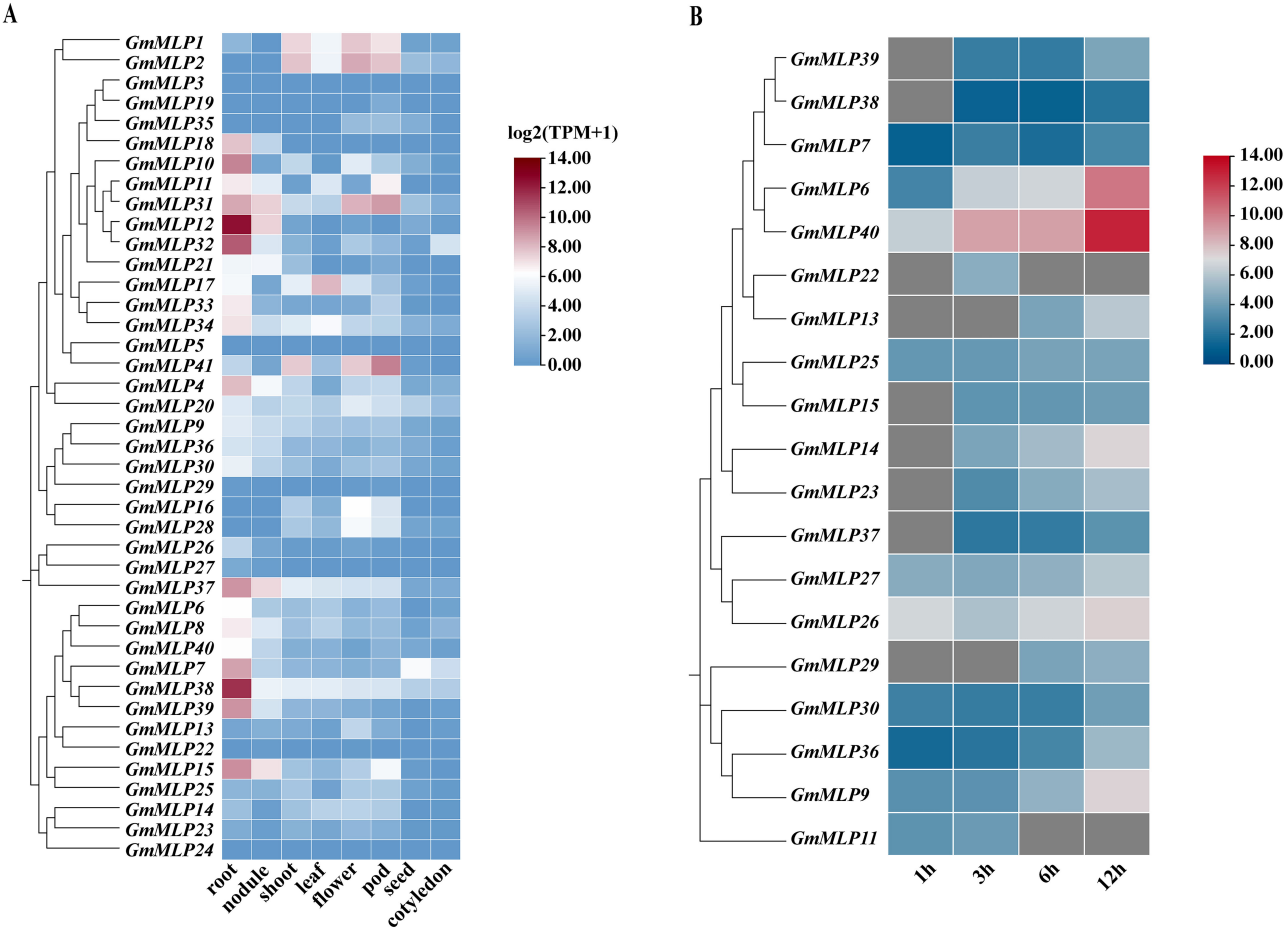
Expression profiles of soybean *GmMLP* genes in different tissues and under salt stress. **(A)** Heatmap showing the expression levels of *GmMLP* genes in different soybean tissues based on public RNA-seq datasets. The expression values are shown as log2(TPM + 1). **(B)** Heatmap showing the expression profiles of salt-responsive *GmMLP* genes in soybean roots based on RNA-seq data generated under salt-stress treatment.

In order to investigate the expression profiles of *GmMLPs* across various tissues, we extracted TPM values of the 41 *GmMLP* genes from the Soybean Expression Atlas v2 and compared their expression levels across roots, leaves, cotyledons, flowers, pods, nodules, and seeds (Almeida-Silva et al., 2023; **Supplementary Table 4**). The 41 *GmMLP* genes exhibited diverse expression profiles across soybean tissues (**Figure 6A**). Notably, the majority of these genes showed elevated expression in roots, with 23 genes, including *GmMLP12*, *GmMLP38*, *GmMLP32*, *GmMLP10*, *GmMLP15*, *GmMLP39*, *GmMLP37*, *GmMLP7*, *GmMLP17* and *GmMLP9*, displaying significantly increased levels. Additionally, several members of the *GmMLP* family exhibited distinct tissue-specific expression in flowers and pods, particularly *GmMLP1*, *GmMLP2*, *GmMLP31*, *GmMLP41*, *GmMLP16*, and *GmMLP28* which were more abundantly expressed in these tissues. In contrast, only a few genes, such as *GmMLP17*, *GmMLP34*, and *GmMLP1*, were primarily expressed in leaves or cotyledons. Although the expression levels of these genes were not the highest in nodules, *GmMLP31*, *GmMLP12*, *GmMLP37*, *GmMLP15*, *GmMLP4*, *GmMLP21*, and *GmMLP38* still exhibited relatively high expression in nodules, suggesting their involvement in root or nodule-related processes in soybeans. Furthermore, genes like *GmMLP3*, *GmMLP5*, *GmMLP24*, *GmMLP22*, and *GmMLP29* displayed low expression across most tissues, indicating that their expression might be limited under standard conditions or could be specific to particular developmental stages or environmental factors.

Through an analysis of transcriptome data from soybean roots subjected to salt stress, previously collected by our research team, we identified significant changes in the expression of 19 *GmMLP* genes at various time points following salt treatment. Furthermore, all these genes were up-regulated at different time points under salt stress. Notably, *GmMLP6*, *GmMLP9*, *GmMLP14*, *GmMLP26*, and *GmMLP40* exhibited particularly pronounced changes in expression levels after salt stress treatment (**Figure 6B**).

### 3.8 Expression profiles of *GmMLP* genes in response to salt stress and ABA treatment

**Figure 7:**
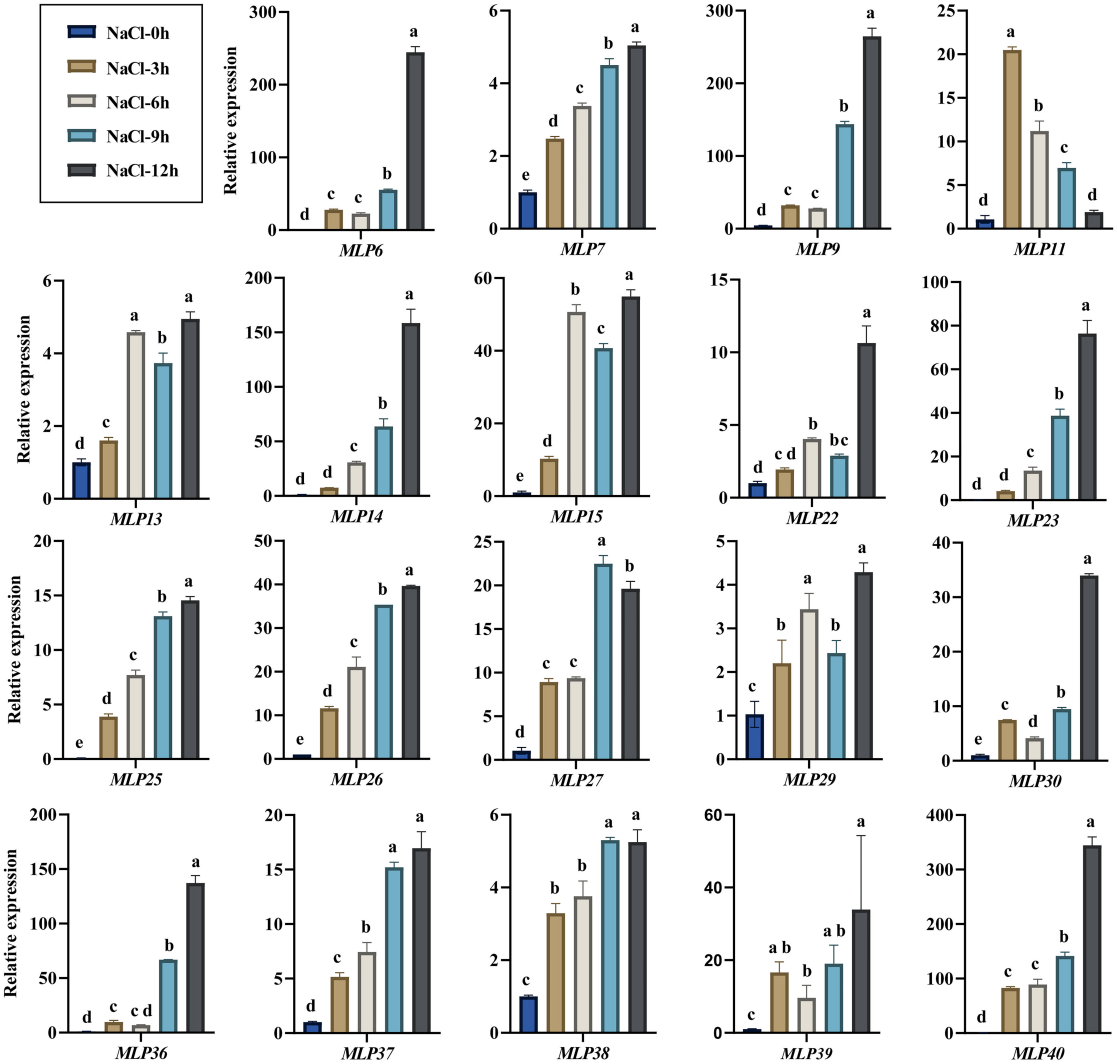
qRT-PCR analysis of selected *GmMLP* genes in soybean roots under salt stress. Relative expression levels of 19 candidate *GmMLP* genes in soybean roots under salt-stress treatment were determined by qRT-PCR. Gene expression was calculated using the 2^−ΔΔCt^ method. Values are means ± SD of three biological replicates. Significant differences between the control and salt-treated samples were determined using One-way ANOVA. Bars with different letters indicate statistically significant difference at *P* < 0.05.

To evaluate the reliability of the transcriptome data, we conducted a repeated salt stress experiment and collected root samples at various intervals for qRT-PCR analysis of 19 selected genes (**Figure 7**). The results demonstrated that the expression of these genes was significantly elevated under stress conditions, aligning with the transcriptome findings. Each of the 19 *GmMLP* genes exhibited notable expression variations at least at one time point following salt exposure. In particular, 14 genes (*GmMLP6*, *GmMLP7*, *GmMLP9*, *GmMLP13*, *GmMLP15*, *GmMLP25*, *GmMLP26*, *GmMLP27*, *GmMLP29*, *GmMLP30*, *GmMLP37*, *GmMLP38*, *GmMLP39*, and *GmMLP40*) demonstrated sustained changes in expression levels as the duration of NaCl treatment increased. Furthermore, seven *GmMLP* genes (*GmMLP6*, *GmMLP9*, *GmMLP14*, *GmMLP23*, *GmMLP36*, *GmMLP39*, and *GmMLP40*) exhibited expression levels exceeding 60 times that of the control at least once, underscoring their robust response to salt stress (**Figure 7**).

**Figure 8:**
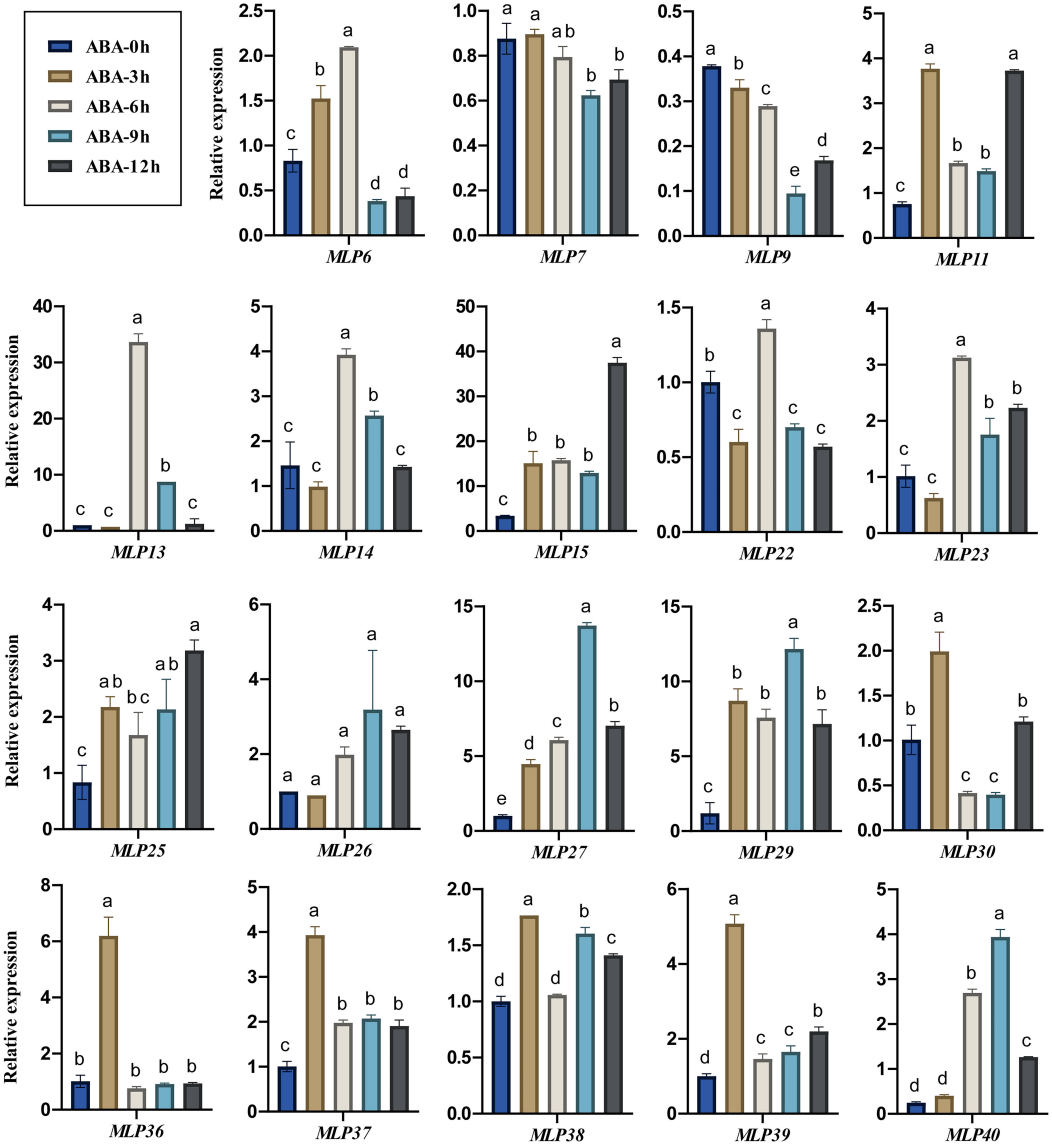
qRT-PCR analysis of selected *GmMLP* genes in soybean roots under ABA treatment. Relative expression levels of 19 candidate GmMLP genes in soybean roots under ABA treatment were determined by qRT-PCR. Gene expression was calculated using the 2^−ΔΔCt^ method. Values are means ± SD of three biological replicates. Significant differences between the control and ABA-treated samples were determined using One-way ANOVA. Bars with different letters indicate statistically significant difference at *P* < 0.05.

We subsequently examined the expression of 19 genes in response to ABA treatment across various time intervals. Our findings revealed that *GmMLP*6, *GmMLP*9, *GmMLP*11, *GmMLP*15, *GmMLP*22, *GmMLP*27, *GmMLP*29, *GmMLP*37 and *GmMLP*39 exhibited significant expression changes at all examined time points under ABA treatment (**Figure 8**). Among these, *GmMLP9* was consistently downregulated at all time points following ABA treatment, while five genes (*GmMLP11*, *GmMLP15*, *GmMLP27*, *GmMLP29*, *GmMLP37*, and *GmMLP39*) were upregulated. Notably, *GmMLP9* demonstrated significant changes at all four time points following ABA treatment (**Figure 8**). Given that *GmMLP9* exhibited substantial variations under both salt stress and ABA conditions, we selected it for further functional validation.

### 3.9 Heterologous expression of *GmMLP9* enhances the tolerance of yeast to salt stress

**Figure 9:**
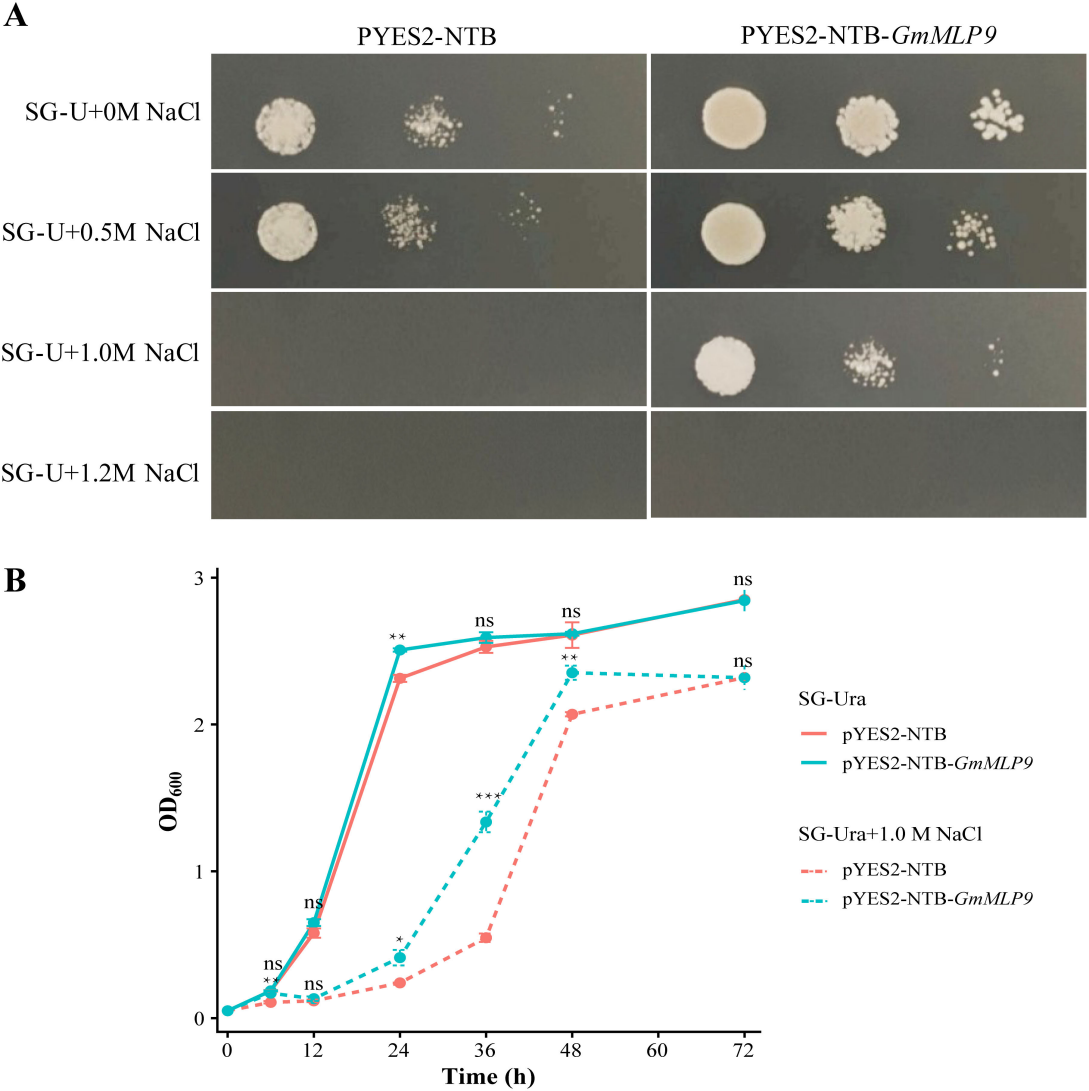
Heterologous expression of *GmMLP9* enhances salt tolerance in yeast. **(A)** Yeast cells carrying the empty vector pYES2-NTB or pYES2-NTB-*GmMLP9* were spotted onto SG-Ura medium supplemented with 0, 0.5, 1.0, or 1.2 M NaCl. Cell suspensions were serially diluted to 10^0^, 10^-1^, and 10–^2^ before spotting. **(B)** OD600 values of yeast liquid cultures at different time points under SG-Ura medium or SG-Ura medium containing 1.0 M NaCl. The data presented in the growth curve are shown as mean ± SD of three independent biological replicates. Significant differences between the control and ABA-treated samples were determined using One-way ANOVA. Bars with different letters indicate statistically significant difference at P < 0.05.

To investigate the role of the *GmMLP9* gene in plant salt response, heterologous expression analysis was performed using a yeast system. Under normal growth conditions, both the empty-vector control strain and the recombinant yeast strain expressing *GmMLP9* exhibited comparable growth, with no significant differences observed acrossamong the various serial dilution gradients. However, when the NaCl concentration in the medium was increased to 1.0 M, distinct growth differences emerged between the recombinant strain and the control strain. Compared to the empty-vector control, yeast cells expressing *GmMLP9* demonstrated markedly improved growth under salt stress conditions. Notably, no visible colony growth was observed in the empty-vector control strain at 1.0 M NaCl, while the *GmMLP9*-expressing strain maintained the ability to grow under the same conditions (**Figure 9A**). Consistent with the spot assay, the liquid growth curve showed that the pYES2-NTB and pYES2-NTB-*GmMLP9* yeast strains exhibited broadly comparable growth under SG-Ura conditions. However, under 1.0 M NaCl, the *GmMLP9*-expressing yeast maintained higher OD600 values than the empty-vector control during the early to middle growth phase, particularly between 24 and 48 h. At 72 h, the difference between the two salt-treated strains diminished (**Figure 9B**). These results suggest that the heterologous expression of *GmMLP9* enhances salt tolerance in yeast under the experimental conditions.

### 3.10 Overexpression of *GmMLP9* reinforces tolerance to salt stress in soybean hairy roots

**Figure 10:**
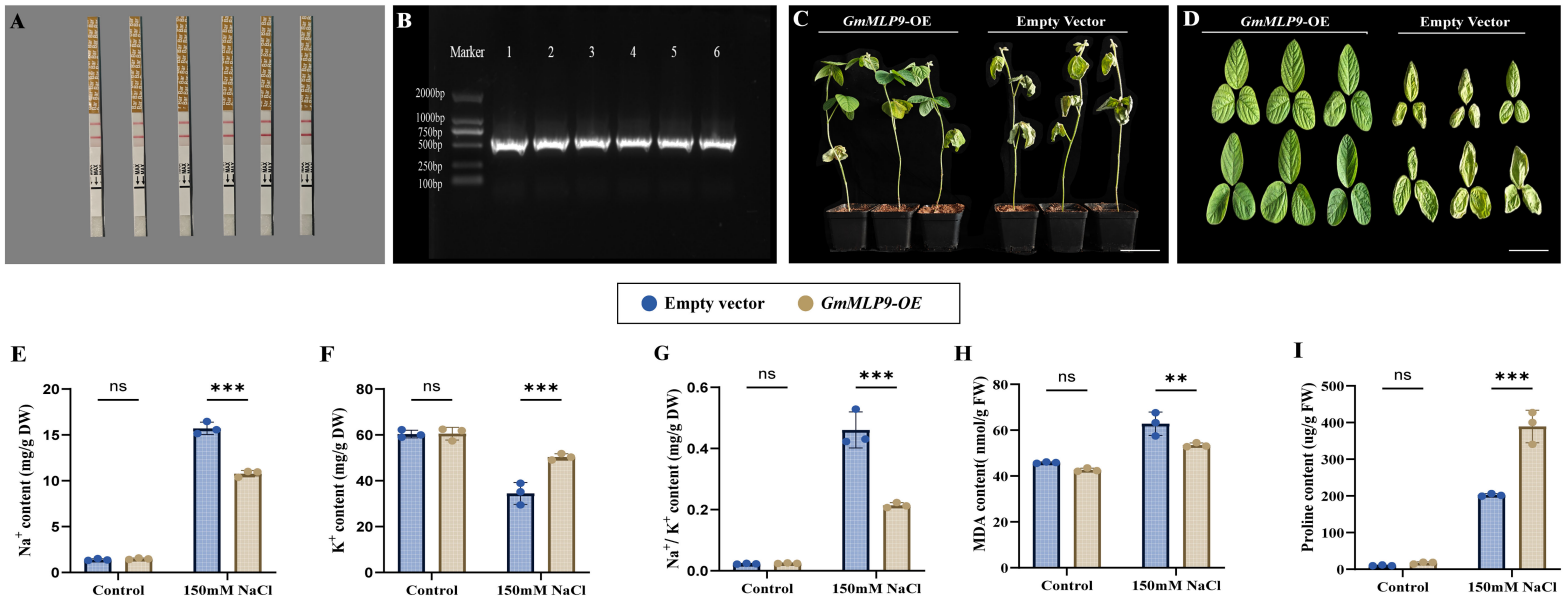
Verification of *GmMLP9*-overexpressing soybean hairy roots and representative phenotypes under salt stress. **(A)** Detection of positive transgenic hairy roots using PAT/bar EPSPS lateral flow dipstick assays. **(B)** PCR verification of positive transgenic hairy roots using Bar-F/R primers. The expected 553 bp amplification product indicates the presence of the transgene. **(C)** Representative growth phenotypes of control and *GmMLP9*-overexpressing soybean hairy-root composite plants under salt-stress conditions. Scale bars=6cm. **(D)** Representative phenotypes of detached trifoliate leaves from control and *GmMLP9*-overexpressing plants under salt-stress conditions. Scale bars=5cm. **(E–I)** Determination of Na^+^ content **(E)**, K^+^ content **(F)**, Na^+^/K^+^ ratio **(G)**, MDA content **(H)**, and proline content **(I)** of control and *GmMLP*-OE hairy roots under normal and 150 mM salt stress. Values are means ± SD of three biological replicates. Significant differences between the control and ABA-treated samples were determined using One-way ANOVA. Bars with different letters indicate statistically significant difference at *P* < 0.05.

To further investigate the role of *GmMLP9* in salt stress responses in soybean, we generated *GmMLP9*-overexpressing hairy roots (*GmMLP9*-OE) in the soybean cultivar QH34 and evaluated their phenotypes under salt stress conditions. Positive transgenic hairy roots were identified using BAR test strips and bar-specific PCR amplification (**Figures 10A, B**). Subsequently, the composite soybean plants were subjected to salt stress treatment for 7 days. The results indicated that the growth of control plants was significantly inhibited under salt stress conditions, as evidenced by pronounced leaf chlorosis, curling, and accelerated wilting. In contrast, *GmMLP9*-OE plants exhibited relatively better growth performance under the same salt treatment conditions, displaying greener leaves and reduced wilting symptoms compared to the control plants (**Figures 10C, D**).

Subsequently, we measured the contents of Na^+^ and K^+^ under both control and salt-stress conditions. Under normal conditions, no significant differences in Na^+^ content, K^+^ content, or the K^+^/Na^+^ ratio were observed between the empty-vector control and the 35S:*GmMLP9* hairy roots. Following NaCl treatment, Na^+^ content increased while K^+^ content decreased in both control and *GmMLP9*-overexpressing hairy roots. However, compared to the empty-vector control, the 35S:*GmMLP9* hairy roots accumulated less Na^+^, maintained higher K^+^ content, and exhibited a lower Na^+^/K^+^ ratio under salt stress (**Figures 10E–G**). Additionally, we measured the contents of MDA and proline. In the control group, there were no significant differences in MDA and proline contents between the empty-vector control and the 35S:*GmMLP9* hairy roots. Under stress conditions, Gm*MLP9*-OE had significantly lower MDA content and higher proline content than those in the empty-vector control (**Figures 10H, I**). Thus, these results demonstrate that overexpression of *GmMLP9* enhances salt tolerance in soybean hairy root composite plants, which is consistent with the results obtained from the yeast heterologous expression assay.

### 3.11 Predicted protein–protein association network of GmMLP9

**Figure 11:**
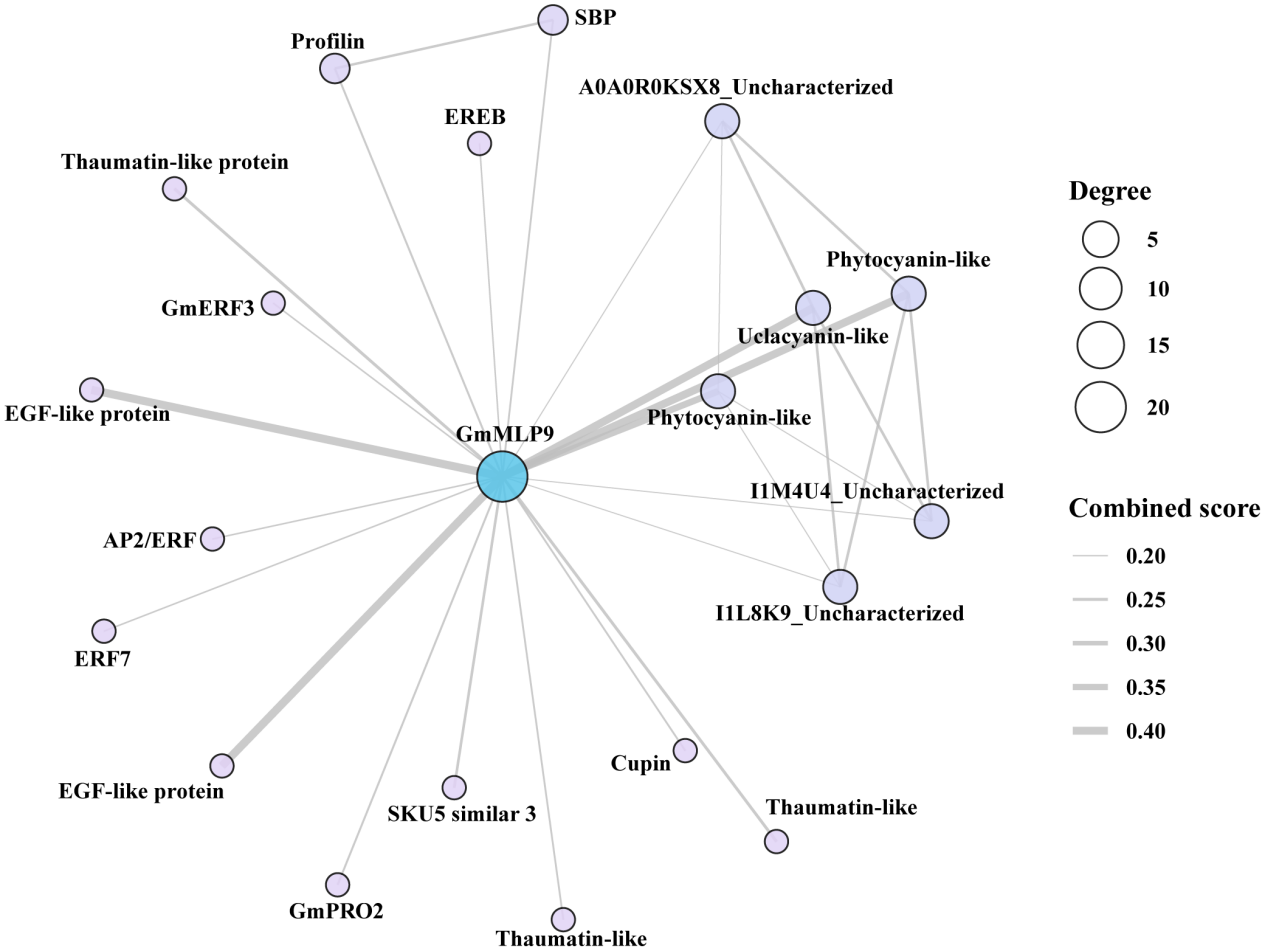
Predicted GmMLP9-associated protein network. The GmMLP9-centered protein association network was constructed using STRING and visualized in R. Each node represents GmMLP9 or one predicted GmMLP9-associated protein, and each edge represents a STRING-predicted protein association. Edge width indicates the STRING combined score, with thicker edges representing higher confidence of association. Node size indicates node degree, defined as the number of edges connected to a given protein in the network.

To investigate the potential connections of GmMLP9 with specific protein modules, we constructed a GmMLP9-centered protein association network using STRING (**Figure 11**; **Supplementary Table 6**). This network comprised 21 nodes and 30 edges, with GmMLP9 exhibiting the highest node degree. The predicted proteins associated with GmMLP9 included stress- and defense-related proteins, such as thaumatin-like proteins and Bet v I/major latex protein-related proteins, as well as AP2/ERF-related transcriptional regulators, including AP2/ERF, EREB, ERF7, and GmERF3. Additionally, several proteins associated with the cell wall or extracellular matrix, such as phytocyanin-like, uclacyanin-like, SKU5-similar 3, EGF-like, and cupin-domain proteins, were identified within the network. These findings suggest that GmMLP9 may be functionally linked to components involved in stress defense and transcriptional regulation; however, these predicted associations necessitate experimental validation.

## 4 Discussion

The *MLP* gene family plays a crucial role in plant growth, development, and stress responses. However, research on soybean *MLP* genes remains limited. In this study, we identified 41 members of the soybean *MLP* family (**Table 1**), which are distributed across 13 of the 20 soybean chromosomes (**Figure 3**). The number of *MLP* genes in soybean significantly differs from that in other species. For instance, *Arabidopsis* has 24 *MLP* genes (Lytle et al., 2009), apple has 36 (Yuan et al., 2020), cucumber has 37 (Kang et al., 2023), peanut has 135 (Li et al., 2023), poplar has 43 (Sun et al., 2024), rose has 46 (Chen et al., 2024), tomato has 34 (Sun et al., 2023), and grape has 14 (Zhang et al., 2018). These findings suggest that the *MLP* gene family has undergone varying degrees of expansion and contraction throughout evolution across various species. Notably, MLP proteins did not cluster by species and were distributed across different clades; some GmMLPs were closely related to SlMLP and AhMLP proteins, indicating conserved homology. Phylogenetic analyses indicate that soybean MLP members are consistently classified into three subcategories (I, II, and IV), highlighting the strong evolutionary conservation of this gene family among various plants (**Figure 1A**). Notably, AhMLP members in peanuts exhibit distinct branches, potentially due to specific tandem duplications and fragments associated with their adaptation to abiotic stressors (Li et al., 2023). Previous studies have reported unique expansion patterns in several species, including cucumber (Kang et al., 2023), tomato (Sun et al., 2023), poplar (Sun et al., 2024), grape (Zhang et al., 2018), apple (Yuan et al., 2020), and rose (Chen et al., 2024).

The expansion of gene families is typically influenced by several processes, including whole-genome duplication, segmental duplication, tandem duplication, and proximal duplication (Lynch and Conery, 2000; Cannon et al., 2004; Qiao et al., 2019). Our analysis of gene duplication and chromosomal mapping revealed 16 pairs of syntenic relationships within the *GmMLP* family, which are distributed across various chromosomes and associated with segmental duplication events (**Figure 4A**). This finding suggests that the proliferation of the soybean *MLP* gene family likely arises from extensive chromosomal segmental duplications. It is essential to recognize that the mechanisms underlying the expansion of the *MLP* family differ significantly among species. For instance, the *MLP* families in cucumber and apple primarily undergo tandem duplication, while those in peanut and poplar exhibit both segmental and tandem duplication (Yuan et al., 2020; Kang et al., 2023; Li et al., 2023; Sun et al., 2024). Prior research has demonstrated that plant genes related to environmental factors and stress responses tend to expand in a species-specific manner, resulting in uneven retention and loss patterns that reduce synteny conservation across species (Hanada et al., 2008). Consequently, the limited collinear relationships observed among species may be attributed to the frequent involvement of *MLP*/*PR-10*-like genes in abiotic stress responses and defense mechanisms (Liu and Ekramoddoullah, 2006). An evaluation of selective pressure revealed that, apart from two pairs of duplicated genes, all other pairs exhibited Ka/Ks ratios below 1, indicating that these genes have mainly undergone purifying selection during evolution. This finding suggests that the coding sequences and their functions are evolutionarily stable, demonstrating resistance to detrimental mutations (Arbiza et al., 2006). This further reinforces the conserved ligand-binding role of the Bet_v_1 domain within the *MLP* gene family. Notably, the Ka/Ks ratios for two pairs of duplicate genes exceeded 1, indicating a potential accumulation of significant non-synonymous substitutions; however, this does not necessarily indicate functional divergence. Considering factors such as low Ks values, the accuracy of sequence alignment, and possible annotation errors, further validation through subcellular localization and transgenic experiments is essential (Yang and Bielawski, 2000; Markova-Raina and Petrov, 2011).

The *GmMLP* gene family exhibits characteristic features typical of Bet_v_1 proteins, particularly in terms of protein size and conserved core domains. Variations in the number and arrangement of conserved motifs among different GmMLP proteins reflect functional differentiation within the same phylogenetic group. Members belonging to the same phylogenetic lineage exhibit significant similarities in exon-intron structures (**Figure 2**), multiple sequence alignments (**Figure 1B**), and motif arrangements (**Figure 2**), suggesting close genetic relationships and potentially analogous biological functions. Most GmMLP proteins possess highly conserved sequence features, with multiple sequence alignment analyses revealing notable conservation in specific regions. Interestingly, certain GmMLP members show exceptionally long sequences, implying possible evolutionary modifications in both their sequences and functions. Genes derived from gene family duplication may retain their essential functions while acquiring mutations in non-core regions, potentially leading to functional specialization. The presence of conserved domains across GmMLP proteins suggests that these members might still share fundamental functions, while highly variable regions could have evolved new biological roles.

Research into gene expression has revealed that different members of the *MLP* family exhibit diverse expression patterns across various tissues (**Figure 6A**). A majority of these genes are predominantly expressed in roots, while others demonstrate increased levels in flowers and pods, suggesting possible functional specialization related to specific tissues within the soybean *MLP* family. Roots serve as the main organ for sensing salt stress, regulating ion uptake, and facilitating long-distance signaling in plants (Zhu, 2002). The highly expressed *GmMLP*s in roots may directly contribute to the initial responses to salt stress, support the stability of root cells, or facilitate the transport of small molecules. Previous studies have indicated that MLP/PR-10 proteins play a role in the formation of floral organs, fruit ripening, and abscission processes (Ruperti et al., 2002; D’Avino et al., 2011; Fujita and Inui, 2021). Therefore, those members with elevated expression in flowers and pods might be linked to reproductive growth or seed development. Some members display relatively low expression across all studied tissues, indicating that they may only be activated during specific developmental phases or in response to stress. Interestingly, a few genes maintain consistently high expression in nodules, suggesting their involvement in nodule-specific metabolic activities or mechanisms for stress adaptation (Gamas et al., 1998; Lepetit and Brouquisse, 2023).

Cis-acting elements located in the promoter region are crucial for the regulating of gene expression. This study revealed that the *GmMLP* promoter contains various elements associated with stress and hormonal responses, including binding sites for transcription factors such as MYB, MYC, and WRKY, as well as hormone-responsive elements for abscisic acid, methyl jasmonate (MeJA), ethylene, salicylic acid, and auxin (**Figure 6**). These findings indicate that transcription factors and plant hormones are involved in regulating the expression of *GmMLP* genes. Previous studies have demonstrated that *MLP* genes contain *WRKY*, *MYB*, and *bZIP* binding sites within their promoters. For instance, *CpWRKY50* can directly bind to the *CpMLP14* promoter and inhibit its expression, thereby participating in the regulation of disease resistance (Yang et al., 2026). In tobacco, *NtWRKY71* regulates the transcription of *NtMLP423* to enhance drought tolerance (Liu et al., 2020). Additionally, the transcription factor *NtMYB108* directly binds to the promoter of *NtMLP423* conferring improved cold stress tolerance (Liu et al., 2023). The zinc finger transcription factor *ZAT5L* participates in wheat drought resistance by inhibiting the *MLP* family member *TaSTP* (Chen et al., 2026). These findings suggest that the expression of *GmMLP* genes may be regulated by these transcription factors in soybean. Future research on the signaling pathways through which *MLPs* regulate plant responses to biotic and abiotic stress should focus on these transcription factor families.

To date, while three *MLP* genes in soybean have been reported, research has primarily focused on their promoter activities and roles in pathogen defense (Crowell et al., 1992; Strömvik et al., 1999; Xu et al., 2014). Currently, there are no reports regarding the involvement of soybean *MLPs* in regulating responses to abiotic stress. In this study, transcriptome screening and RT-qPCR validation demonstrated that the expression of 19 candidate *GmMLP*s is regulated by salt stress (**Figure 6B**). Furthermore, we identified that seven *GmMLPs* were continuously regulated by both salt and ABA (**Figure 7**, **8**). Among these, *GmMLP9*, which showed sensitivity to NaCl and ABA, was selected for further analysis through ectopic expression in yeast (**Figure 9**) and overexpression in soybean (**Figure 10**). Previous studies have indicated that MLPs mitigate salt stress damage via multiple strategies, including maintaining ROS balance, stabilizing cell membranes, and facilitating the accumulation of osmotic adjustment compounds (Cutler et al., 2010; Yoshida et al., 2014; Sah et al., 2016; Wang et al., 2016; Liu et al., 2023). In this study, heterologous expression of *GmMLP9* conferred enhanced salt tolerance in yeast under saline conditions, as evidenced by superior cell growth (**Figure 9**). Consistently, overexpression of *GmMLP9* in soybean hairy roots reduced the Na^+^/K^+^ ratio under salt treatment (**Figures 10E–G**), indicating that GmMLP9 positively modulates salt tolerance by maintaining cellular ion homeostasis. Proline acts as a core compatible solute that facilitates osmotic adjustment and protects plant cells from damage caused by abiotic stress (Alvarez et al., 2022). Meanwhile, MDA serves as a classic marker indicating the extent of membrane lipid peroxidation and oxidative injury (Alché, 2019). In this study, we found that salt-stressed soybean hairy roots overexpressing *GmMLP9* accumulated lower levels of MDA (**Figure 10H**) and higher levels of proline (**Figure 10I**) compared to the control group. Collectively, these physiological data suggest that *GmMLP9* functions as a positive regulator of salt tolerance in soybean by coordinating ion balance and alleviating oxidative damage.

As a key hormone in the response to salt stress, ABA regulates the expression of numerous stress-responsive genes to mediate plant tolerance to abiotic stresses (Lee and Luan, 2012). Previous studies have demonstrated that the *MLP* gene is involved in the regulation of abiotic stress responses through both ABA-dependent and ABA-independent pathways (Wang et al., 2016; Liu et al., 2020). In this study, the expression of the *GmMLP9* gene exhibited contrasting expression patterns under salt and ABA treatments. Therefore, we hypothesize that *GmMLP9* may positively regulate soybean salt tolerance through an ABA-independent pathway. Results from protein association network analysis indicated that GmMLP9 is linked to proteins associated with stress defense, AP2/ERF-type transcriptional regulation, and extracellular/cell-wall-related processes (**Figure 11**; **Supplementary Table 6**). Additionally, cotton GhMLP28 has been shown to interact with GhERF6, promoting the specific binding of GhERF6 to the GCC-box, thereby enhancing cotton disease resistance (Yang et al., 2015). This supports a potential connection between MLP proteins and ERF-related regulatory modules. In conjunction with the analysis of cis-acting elements in the promoters of *GmMLPs*, we speculate that under salt stress, transcription factors such as MYB and WRKY may bind to the promoters of *MLP* genes to regulate their expression. Additionally, MLPs might interact with transcription factors like ERFs, influencing their binding to downstream genes to regulate soybean salt tolerance. This regulatory mechanism warrants further verification through experimental studies in the future. Furthermore, the functional identification of *GmMLP9* was conducted exclusively using a hairy root transformation system, as stable transgenic soybean lines corresponding to this gene are currently unavailable. Therefore, the function of the *GmMLP9* gene cannot be verified in field conditions. Future research will focus on constructing stable transgenic soybean lines to further validate the endogenous role of *GmMLP9* in response to salt stress.

## 5 Conclusions

This study provided a comprehensive characterization of the MLP family members in soybean, demonstrating that their expansion primarily occurred through segmental duplication and that they have predominantly undergone purifying selection over an extended evolutionary period. Through phylogenetic analysis, synteny assessments, identification of promoter elements, tissue expression studies, NaCl/ABA response tests, and functional validation, we identified significant functional divergence within this family. Notably, GmMLP9 appears to play a critical role as a positive regulator in enhancing root tolerance to salt stress. This study establishes a vital genomic foundation for a deeper understanding of the potential roles of this family in the response to salt stress and hormonal regulation in soybeans.

## Data Availability

The datasets presented in this study are deposited in the NCBI Sequence Read Archive, accession number (BioProject: PRJNA1482683).
